# *Santocellus* (Neuroptera, Chrysopidae, Leucochrysini): taxonomic changes, new description, and a key to the species

**DOI:** 10.3897/zookeys.255.4111

**Published:** 2012-12-28

**Authors:** Catherine A. Tauber

**Affiliations:** 1 Department of Entomology, Comstock Hall, Cornell University, Ithaca, NY 14853-2601, and Department of Entomology, University of California, Davis, CA

**Keywords:** *Santocellus*, *Leucochrysa*, Neotropical, Leucochrysini, Peru

## Abstract

*Santocellus* is a small Neotropical genus of leucochrysine lacewings that only recently was separated from *Leucochrysa*. Here, the features of the *Leucochrysa risi* Esben-Petersen holotype (a female) are described and shown to support the species’ transfer to *Santocellus* and the continued retention of the genus *Santocellus* as separate from *Leucochrysa*. The valid name for the species becomes *Santocellus risi* (Esben-Petersen, 1933), **comb. n.**, and *Santocellus bullata* (Tauber, 2007) is recognized as a **syn. n.** of *Santocellus risi*. Currently, this species is reported only from Peru. An illustrated key is provided for distinguishing the known species in the genus *Santocellus*.

## Introduction

The Neotropical genus *Santocellus* (Neuroptera: Chrysopidae, Leucochrysini) was recently differentiatied from other leucochrysine genera on the basis of a subtle, but consistent, suite of adult and larval traits (Tauber et al. 2008). Currently, the genus contains three species, all from South America: *Santocellus atlanticis* Tauber & Albuquerque, *Santocellus bullata* Tauber, and *Santocellus riodoce* Tauber. One of the species, *Santocellus bullata* previously was known only from a single male collected in Peru – the holotype at the National Museum of Natural History, Washington DC (USNM) (Tauber 2007). During a recent visit to the Museum of Comparative Zoology (MCZ), a female specimen of this species was discovered; it also was from Peru. However, it had been identified by P. A. Adams as *Leucochrysa risi* Esben-Petersen. Subsequent examination of the *Leucochrysa risi* type [= *Santocellus risi* (Esben-Petersen)] in the Zoological Museum of Copenhagen University (ZMCU) confirmed Adams’ identification and the synonymy of *Santocellus bullata* and *Leucochrysa risi*.

Herein, the features of the previously unknown female are described and illustrated; the results are used to examine the consistency of the female features among *Santocellus* species. In addition, a key with illustrations for identifying *Santocellus* species is provided. Methods for staining the abdomen and making measurements were those used previously (see Tauber 2007).

## Taxonomy

### Santocellus risi

(Esben-Petersen, 1933) comb. n.

http://species-id.net/wiki/Santocellus_risi

**Figure 1.**
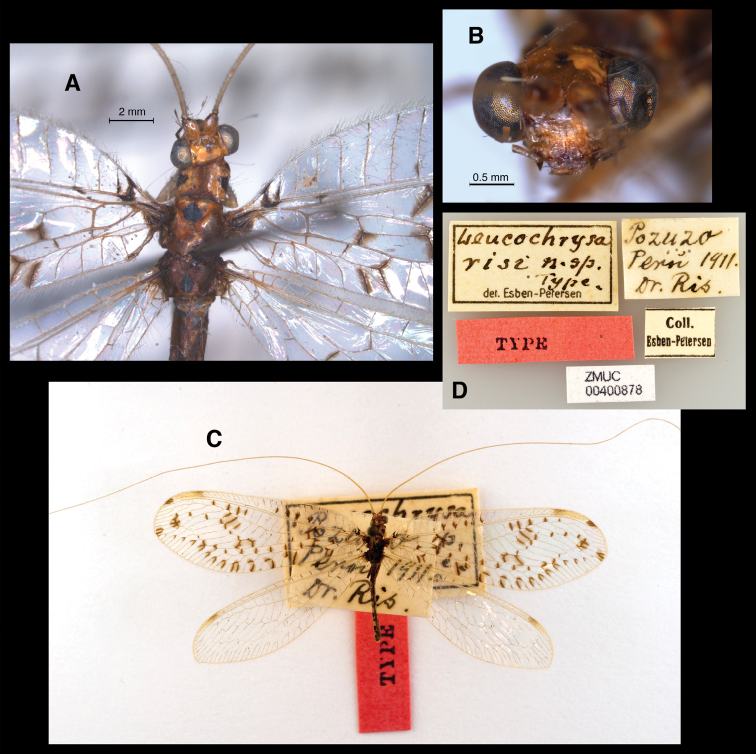
*Leucochrysa risi* Holotype [Female, Pasco, Peru; Zoological Museum of Copenhagen University; Photos by Niels P. Kristensen] **A** Head, thorax, dorsal **B** Head, frontal **C** Habitus, dorsal **D** Labels.

Figs 1 [Fig F2] 3 4C, 4D 5E, 5F

**Figure 2. F2:**
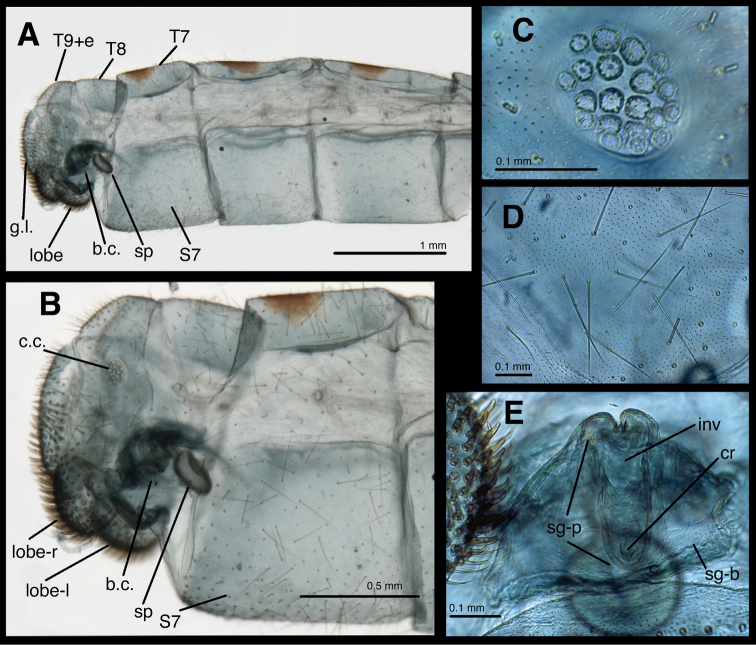
Female abdomen of *Leucochrysa risi* [Female (mature), Pasco, Peru]. **A** Segments A5 to A9+ectoproct, lateral **B** Segments A7 to A9+ectoproct, lateral **C** Callus cerci (trichobothria missing) **D** Abdominal integument **E** Subgenitale, posterior. Abbreviations: **b.c.** bursa copulatrix; **c.c.** callus cerci; **cr** crumena of subgenitale; **g.l.** gonapophysis lateralis; **inv**, invagination below distal lobes of subgenitale; **lobe** setose lobe at ventral margin of ectoproct; **lobe-l** lobe on left side of body; **lobe-r** lobe on right side of body; **sg-b** base of subgenitale; **sg-p** elongate ventral process of subgenitale; **sp** spermatheca; **S7** seventh sternite; **T7,** **T8** seventh and eighth tergites; **T9+e** fused ninth tergite and ectoproct.

Leucochrysa risi Esben-Petersen, 1933: 119 [ZMCU, Holotype (by original designation), Figs 1–2; original description: “One specimen Pozuzo, Peru”]. Penny 1977: 23 [species list]; Núñez 1989: 70 [species list].
Leucochrysa (Nodita) risi Esben-Petersen. Listed in Oswald 2007 (previous publication of the name not confirmed).
Leucochrysa (Leucochrysa) risi Esben-Petersen. Brooks and Barnard 1990: 277 [subgeneric assignment, species list].
Leucochrysa bullata Tauber, 2007: 128 [USNM, Holotype (by original designation), Figs 4C, 5E, F; original description: “Peru. Madre de Dios: Manu: Aguajal, 5 km. S. Pakitza (12°7'S, 70°58'W) 250 m, 18-19-IX-1988, Flint & Erwin”]. Oswald 2007.
Santocellus bullata Tauber et al., 2008: 315 [transfer to *Santocellus*] **syn. n.**

#### Description of female.

*Head, thorax, wings* (Figs 4C, 4D, 5E, 5F). Same as described for male (Tauber 2007, as *Leucochrysa bullata*).

**Figure 3.**
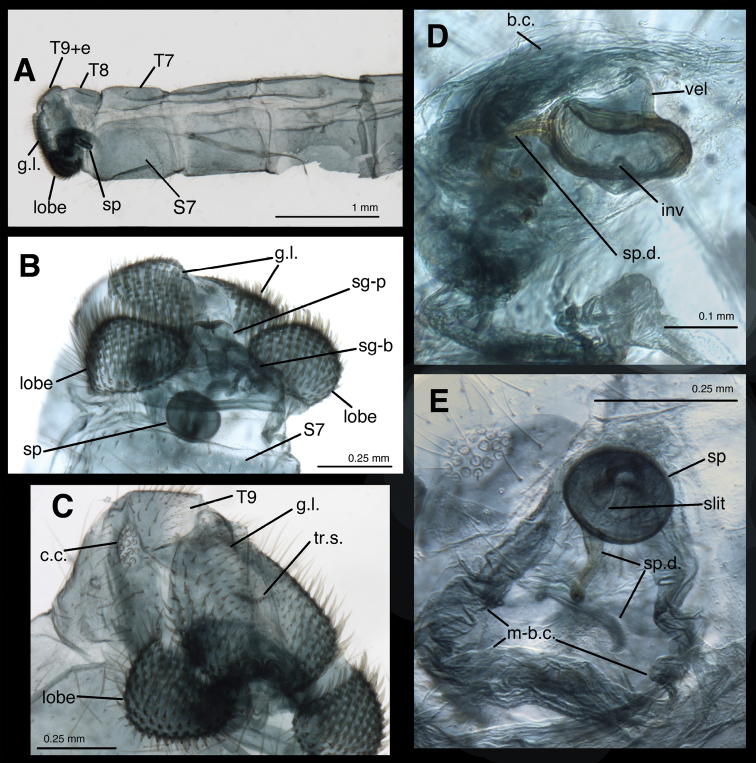
Female abdomen of *Leucochrysa risi* [Female (slightly teneral), Junin, Peru] **A** Segments A5 to A9+ectoproct, lateral **B** Abdominal terminus, posteroventral, showing enlarged, setose lobes on ventral margin of ectoproct. Note subgenitale nestled between left and right lobes, round spermatheca (with dorsal slit) above subgenitale **C** Abdominal terminus, ventrolateral, showing enlarged, setose lobes on ventral margin of ectoproct, setose surface of gonapophysis lateralis **D** Spermatheca below bursa copulatrix, lateral view **E** Spermatheca and spermathecal duct engulfed within membrane of bursa copulatrix, ventral view. Abbreviations: **b.c.** bursa copulatrix; **c.c.** callus cerci; **g.l.** gonapophysis lateralis; **inv** spermathecal invagination; **lobe** setose lobe on ventral margin of ectoproct; **m-b.c.** membrane of bursa copulatrix; **sg-b** base of subgenitale; **sg-p** elongate ventral process of subgenitale; **slit** slit in dorsal surface of spermathecal velum, opening to bursal duct above (not visible); **sp** spermatheca; **sp.d.** spermathecal duct; **S7** seventh sternite; **T7, T8** seventh and eighth tergites; **T9+e** fused ninth tergite and ectoproct; **tr.s.** transverse sclerite; **vel**, spermathecal velum.

*Female abdomen* (Figs 1C, 2, 3). Segments 1–7 long, slender; tergites shallow [ratio length : width = 8.6 (T5), 7.5 (T6), 4.0 (T7)], with slightly rounded margins, with brown circular spot mesally; T8 shorter, rounded, without brown spot. Sternites deep [ratio length : width = 1.5 (S5), 1.0 (S6), 1.4 (S7)], with dorsal margins slightly depressed (concave) mesally; tergites, sclerites with numerous, long, thin setae, dense microsetae, without microtholi. Pleural region with microsetae, P7 with long, thin setae; spiracles small, simple, with unenlarged atria.

*Female genitalia*. Callus cerci round to slightly oval, 0.10–0.15 mm in diameter, with 19–21 relatively thin trichobothria (longest ~0.13 mm long); cupuliform bases of variable diameter. Tergite 9 + ectoproct rounded, fused dorsally, blunt posteriorly, elongate, ventral section on each side enlarged into pair of bulbous lobes extending well below gonapophyses laterales; enlargement covered with dense, stout, upward-curving setae. Gonapophysis lateralis not large, occupying approximately one-half of posterior margin of abdomen; surface covered with robust, stout setae, especially on ventral half; interior membranous area not greatly expanded. Colleterial gland transparent, delicate, ovoid, small, mostly within gonapophyses laterales and T9+ect, not extending anteriorly much beyond bursa, but with numerous elongate accessory glands attached distally, with transparent, membranous tubule connecting to small reservoir; transverse sclerification short, narrow, receiving short duct from reservoir. Entire genital structure small, not much larger than subgenitale. Bursa copulatrix membranous, broad basally (near subgenitale), tapering and extending slightly into region above S7, folded dorsally, with slight longitudinal depression dorsally, connected ventrally to spermatheca via elongate dorsal slit on spermathecal velum and bursal duct at proximal tip of velum. Bursal duct very short, slender. Bursal glands not seen. Spermatheca doughnut shaped, tucked within distal end of bursa, with small, sail-like velum dorsally, small, V-shaped invagination ventrally. Spermathecal duct attached dorsally to distal end of spermatheca, short, sclerotized, extending into and out of subgenitale, with ~three curves, closely attached to membranes of bursa and subgenitale; terminus with long, dense setae. Subgenitale broad basally, rounded distally, nestled between ventral lobes of ectoproct, narrow in lateral view, with shallow ventral fold at attachment to S7, slightly deeper fold above, terminal process flat, long, extending almost full length of subgenitale, with pair of lobes at base, shallow crumena at rounded tip; membrane above subgenitale with crescent-shaped, lightly sclerotized lamellae.

#### Specimens examined.

Holotype (ZMCU) and a second female specimen (MCZ), with labels reading: [1] “El Campamiento Col. P?r?n? [“?” mine] PERU 1 July ’20”, [2] “Cornell Univ. Expedition Lot 569”, [3] “Leucochrysa (or Nodita) risi Esb-Petersen 1932 det. P.Adams 1974”. The locality data appear to refer to the Expedition’s Camp at Perené in the province of Chanchamayo, Junin, Peru, elevation 696 m (Cornell University Insect Collection Voucher Lot Series, Lot 569).

#### Known distribution.

Currently, this species is known only from three regions of Peru: Junin (~650 m) (new record), Pasco (~800 m) (Esben-Petersen 1933), and Madre de Dios (250 m) (Tauber 2007).

#### Comparison with other *Santocellus* species.

The genus *Santocellus* was described on the basis of a distinctive suite of larval and adult (male and female) character states. However, *Santocellus risi* (as *bullata*) was included in the genus only on the basis of its male characteristics (Tauber et al. 2008); both the female and the larvae were unknown when the genus was described. We now know that, in addition to the striking pustulate wings and unusual body markings that typify the species, *Santocellus risi* females have abdominal characteristics that are distinctive among the Leucochrysini (setose, bulbous lobes on the ventral margins of the ectoproct and unique, stout, curved setae). However, they also share a large set of female features with their congeners, *Santocellus atlanticis* and *Santocellus riodoce*: (1) a round, pillbox-shaped spermatheca with a shallow invagination; (2) a relatively short, lightly sclerotized spermathecal duct; (3) spermatheca with a sail-like velum that opens via a slit to a short bursal duct; (4) spermathecal/bursal complex relatively small, spermatheca nestled below the bursa copulatrix; (5) gonapophyses laterales relatively round and short; (6) colleterial gland bulbous, delicate, transparent, and with several elongate tubules attached to the distal end; (7) subgenitale with two, small to medium-sized, basal folds (at the attachment to the seventh sternite), with a ventral process that is elongate and flat, has a rounded distal margin, rounded lobes basally, and a shallow crumena. The expression of this set of features by the female of *Santocellus risi* provides new support for keeping the genus *Santocellus* separate from *Leucochrysa*, and it offers strong evidence for retaining the species within the genus.

**Figure 4.**
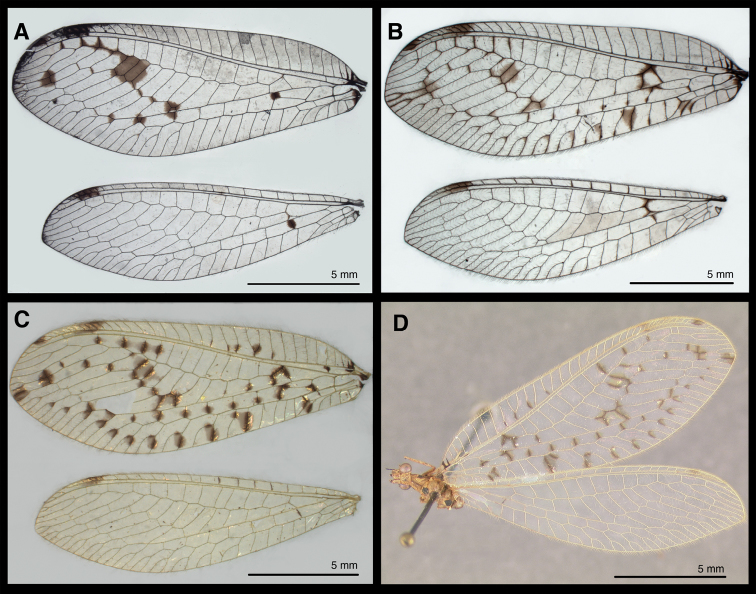
Wings of known *Santocellus* species. **A** *Santocellus atlanticis* [Female; Rio de Janeiro, Brazil] **B** *Santocellus riodoce* [Female; Espírito Santo, Brazil] **C** *Santocellus risi* [Male; Madre de Dios, Peru] **D** *Santocellus risi* [Female; Junin, Peru].

**Figure 5.**
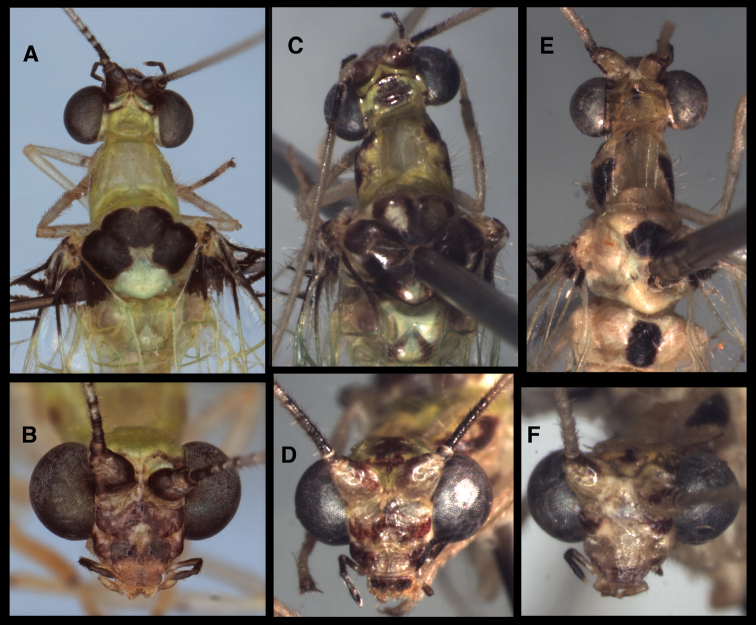
External features of known *Santocellus* species. Top row: Head and thorax, dorsal; Bottom row: Head, frontal **A, B** *Santocellus atlanticis* [Male; Rio Grande do Sul, Brazil] **C, D** *Santocellus riodoce* [Female; Espírito Santo, Brazil] **E, F** *Santocellus risi* [Male; Madre de Dios, Peru].

##### Key to *Santocellus* Species

The key below is intended for identification without dissecting the specimens. For species-specific differences in male and female terminalia, see Tauber 2007, Tauber et al. 2008, Figs 2 and 3 above).

**Table:** 

1	Membrane surrounding numerous crossveins of forewing with pustulate swellings (Figs 4C, 4D); meso- and metanotum each with large, dark brown to black, mesal spot (Fig. 5E)	*Santocellus risi* (Esben-Petersen)
–	Membrane of forewing smooth, without swellings (Figs 4A, 4B); mesonotum either largely or entirely dark brown to black, with or without light green areas (Figs 5A, 5C)	2
2	Forewing with cells between Radial sector (Rs) and inner gradates 5-6 entirely filled with brown, Rs and all crossveins between Rs and Psm without dark clouding (Fig. 4A); mesoscutellum light green (Fig. 5A)	*Santocellus atlanticis* Tauber & Albuquerque
–	Forewing with cells between Radial sector (Rs) and inner gradates 5-6 only partially filled with brown, Rs and first two crossveins between Rs and Psm with dark clouding (Fig. 4B); mesoscutellum largely brown, posterior with small light green spot (Fig. 5C)	*Santocellus riodoce* Tauber

## Supplementary Material

XML Treatment for Santocellus risi

## References

[B1] Brooks SJ Barnard PC (1990) The green lacewings of the world: a generic review (Neuroptera: Chrysopidae). Bulletin of the British Museum of Natural History (Entomology) 59: 117–286

[B2] Esben-Petersen P (1933) New and little-known Neuroptera. Videnskabelige Meddelelser fra Dansk Naturhistorisk Forening, Kobenhaven 94: 109-123

[B3] Núñez E (1989) Chrysopidae (Neuroptera) del Perú y sus especies más comunes. Revista Peruana de Entomologia 31: 69-75

[B4] Oswald JD (2007) Neuropterida Species of the World. Version 2.0. http://lacewing.tamu.edu/species-catalogue/ [Last accessed October 1, 2012]

[B5] Penny ND (1977) Lista de Megaloptera, Neuroptera e Raphidioptera do México, América Central, ilhas Caraíbas e América do Sul. Acta Amazonica 7(4)(Suplemento): 1–61

[B6] Tauber CA (2007) Review of *Berchmansus* and *Vieira* and description of two new species of *Leucochrysa* (Neuroptera: Chrysopidae). Annals of the Entomological Society of America 100: 110-138 doi: 10.1603/0013-8746(2007)100[110:ROBAVA]2.0.CO;2

[B7] Tauber CA Tauber MJ Albuquerque GS (2008) A new genus and species of green lacewings from Brazil (Neuroptera: Chrysopidae: Leucochrysini). Annals of the Entomological Society of America 101: 314-326 doi: 10.1603/0013-8746(2008)101[314:ANGASO]2.0.CO;2

